# Association of paternal age with embryological outcomes and β-hCG positivity in women with polycystic ovary syndrome undergoing ICSI: a retrospective cohort study

**DOI:** 10.3389/fmed.2026.1915304

**Published:** 2026-09-09

**Authors:** Fırat Şahin, Mustafa Kemal Özel, Mehmet Uğur Karabat, Zuhal Çankırı, Fırat Aşır, Mehmet Turan Çetin

**Affiliations:** 1 Division of Infertility, Turan Çetin in-vitro Fertilization Center, Çukurova, Adana, Türkiye; 2 Department of Histology and Embryology, Medical Faculty, Dicle University, Diyarbakır, Türkiye

**Keywords:** embryo development, fertilization, ICSI, intracytoplasmic sperm injection, paternal age, pcos, polycystic ovary syndrome, β-hCG

## Abstract

**Background:**

The independent effect of paternal age on assisted reproductive technology outcomes remains uncertain, particularly when maternal characteristics and conventional semen parameters are considered. This study investigated the association between paternal age and embryological outcomes and β-hCG positivity in women with polycystic ovary syndrome (PCOS) undergoing intracytoplasmic sperm injection (ICSI).

**Methods:**

This single-center retrospective cohort study included 192 ICSI cycles in women with PCOS aged ≤35 years. Male partners were categorized as <35 years (*n* = 129), 35–39 years (*n* = 44), or ≥40 years (*n* = 19). Embryological outcomes included the numbers of retrieved oocytes, metaphase II (MII) oocytes, two-pronuclear (2PN) embryos, cleavage-stage embryos, and cryopreserved embryos, as well as fertilization, cleavage, and cryopreservation rates. β-hCG positivity was evaluated as the early pregnancy outcome. Multivariable logistic regression was used to assess the association between paternal age and β-hCG positivity after adjustment for female age, male and female body mass index, male smoking and alcohol use, baseline anti-Müllerian hormone, pre-OPU estradiol, sperm concentration, and sperm motility.

**Results:**

Maternal age differed significantly among paternal age groups (*p* < 0.001), whereas sperm concentration and motility and other evaluated baseline characteristics were comparable. The numbers of retrieved oocytes, MII oocytes, 2PN embryos, cleavage-stage embryos, and cryopreserved embryos did not differ significantly among paternal age groups. Cleavage and cryopreservation rates were also comparable. Fertilization rate showed an overall between-group difference (*p* = 0.044); however, none of the pairwise comparisons remained significant after Bonferroni correction. β-hCG positivity was 63.6%, 45.5%, and 52.6% in the <35, 35–39, and ≥40-year groups, respectively (*p* = 0.095). In multivariable analysis, paternal age was not independently associated with β-hCG positivity (overall *p* = 0.358).

**Conclusion:**

Paternal age was not independently associated with β-hCG positivity or with a consistent deterioration in embryological outcomes among women with PCOS undergoing ICSI. Although an overall difference in fertilization rate was observed, this finding was not confirmed by corrected pairwise comparisons. These results should be interpreted cautiously because of the small ≥40-year subgroup and the absence of later pregnancy outcomes and molecular sperm-quality assessments.

## 1 Introduction

Polycystic ovary syndrome (PCOS) is a common endocrine disorder and an important cause of infertility among women of reproductive age (1). In women undergoing assisted reproductive technology (ART), the endocrine and metabolic alterations associated with PCOS may influence follicular development, oocyte competence, and reproductive outcomes (1, 2). Although maternal characteristics remain major determinants of ART success, increasing attention has been directed toward the contribution of paternal factors to fertilization and embryo development (3).

Advanced paternal age has been associated with age-related changes in spermatogenesis, including increased oxidative stress, impaired sperm DNA integrity, accumulation of *de novo* mutations, and epigenetic alterations (4, 5). Importantly, these molecular changes may occur even when conventional semen parameters remain within normal reference ranges. Therefore, normal sperm concentration and motility may not completely exclude age-related deterioration in sperm functional or genetic competence (6, 7).

However, the effect of paternal age on ART outcomes remains controversial. Some studies have reported associations between increasing paternal age and impaired fertilization, embryo development, blastocyst quality, or pregnancy outcomes, whereas others have found no independent effect after accounting for maternal and reproductive characteristics (5, 8, 9). Interpretation of previous findings is further complicated by differences in paternal age thresholds, study populations, semen characteristics, and maternal confounders.

Evaluating paternal age among normospermic men and within a relatively homogeneous female population may help reduce some of these sources of heterogeneity (10–12). Women younger than 35 years with PCOS provide a clinically defined population in which the potential contribution of paternal age can be examined while limiting the strong confounding effect of advanced maternal age (13–15). Nevertheless, relevant maternal and paternal characteristics should also be considered when assessing independent paternal-age effects.

Therefore, this study aimed to investigate the association between paternal age and embryological and early pregnancy outcomes in ICSI cycles involving women younger than 35 years with PCOS and normospermic male partners. Paternal age was evaluated in three groups (<35, 35–39, and ≥40 years), and outcomes included oocyte and embryo numbers, fertilization, cleavage and cryopreservation rates, and β-hCG positivity. We hypothesized that increasing paternal age could adversely affect embryological outcomes even in the absence of overt abnormalities in conventional semen parameters.

## 2 Materials and methods

### 2.1 Ethical approval

This retrospective cohort study was conducted in accordance with the principles of the Declaration of Helsinki. Ethical approval was obtained from the Çukurova University Faculty of Medicine Research Ethics Committee (Meeting No. 161, Decision No. 60, December 5, 2025). Written informed consent was obtained from all participants.

### 2.2 Study design and patient population

This single-center retrospective cohort study was conducted at Prof. Dr. M. Turan Çetin IVF Center. Medical, clinical, and embryological records of women with polycystic ovary syndrome (PCOS) who underwent intracytoplasmic sperm injection (ICSI) treatment between January 2020 and August 2025 were retrospectively reviewed. A total of 192 eligible ICSI cycles with complete clinical and embryological data were included in the final analysis. PCOS was diagnosed according to the Rotterdam criteria (16). The diagnosis required the presence of at least two of the following three features after exclusion of other etiologies that could mimic PCOS: (1) oligo- and/or anovulation, (2) clinical and/or biochemical hyperandrogenism, and (3) polycystic ovarian morphology on ultrasonography. The diagnosis of PCOS had been established before initiation of the ICSI cycle and was verified from the patients' medical records during retrospective data collection.

To reduce the confounding influence of maternal age on oocyte and embryo development, the study population was restricted to women younger than 35 years. Eligible cycles were required to involve women with PCOS undergoing ICSI and to have complete clinical, ovarian stimulation, semen analysis, and embryological records. Exclusion criteria included female age ≥35 years, BMI <18 or >35 kg/m^2^, relevant chronic systemic disease, endometriosis, tubal-factor infertility, major uterine abnormalities, cycles involving preimplantation genetic testing, and incomplete data required for the predefined study outcomes. For the primary analyses, male partners were categorized into three paternal age groups: <35 years, 35–39 years, and ≥40 years. Because no universally accepted cutoff for advanced paternal age exists, these categories were used to evaluate potential age-related differences across clinically interpretable paternal age strata, with ≥40 years representing the advanced paternal-age category. The <35-, 35–39-, and ≥40-year groups comprised 129, 44, and 19 cycles, respectively. Paternal age was also examined as a continuous variable, and an additional analysis comparing men aged <40 and ≥40 years was performed to evaluate the findings using the 40-year threshold.

Clinical and laboratory variables extracted from the medical records included maternal and paternal age, duration of infertility, maternal and paternal body mass index (BMI), male smoking and alcohol use, baseline anti-Müllerian hormone (AMH), pre-oocyte retrieval (pre-OPU) serum estradiol (E2), sperm concentration, and sperm motility. These variables were evaluated as baseline characteristics and, where appropriate, as potential confounding factors in subsequent analyses.

### 2.3 Controlled ovarian stimulation and oocyte retrieval

Controlled ovarian stimulation was performed using a gonadotropin-releasing hormone (GnRH) antagonist protocol. Follicular development was monitored by serial transvaginal ultrasonography and serum estradiol (E2) measurements. Final oocyte maturation was triggered with a GnRH agonist when at least three follicles reached a mean diameter of ≥17 mm (17). Serum E2 levels obtained before oocyte retrieval (pre-OPU E2) were recorded for the study analyses. Transvaginal ultrasound-guided oocyte retrieval was performed approximately 36 h after triggering. Retrieved cumulus–oocyte complexes were denuded, and oocyte nuclear maturation was assessed. Oocytes at the metaphase II (MII) stage were identified and used for intracytoplasmic sperm injection (ICSI). The total number of retrieved oocytes and the number of MII oocytes were recorded for each cycle.

### 2.4 ICSI procedure and embryo culture

Semen parameters recorded as part of the routine ART evaluation were retrospectively obtained from the patients' medical records. Sperm concentration (×10⁶/mL) and motility (%) were included in the present analyses. ICSI was performed using MII oocytes according to standard laboratory procedures. Fertilization was evaluated 16–18 h after microinjection and was defined by the presence of two pronuclei (2PN). The number of normally fertilized 2PN oocytes was recorded for each cycle. Embryonic development was subsequently monitored during culture. Cleavage-stage development was assessed during the early culture period, and embryos were cultured up to Day 5 according to the center's routine laboratory protocol. The numbers of cleaved and cryopreserved embryos were recorded for each cycle.

### 2.5 Outcome measures

The primary clinical outcome was β-hCG positivity, defined according to the post-treatment serum β-hCG result recorded in the clinical database. Embryological outcomes included the total number of retrieved oocytes, number of MII oocytes, number of normally fertilized 2PN embryos, fertilization rate, number of cleaved embryos, cleavage rate, number of cryopreserved embryos, and cryopreservation rate. β-hCG positivity was additionally evaluated across paternal age groups and used as the dependent variable in the multivariable logistic regression analysis. The developmental rates were calculated from the corresponding cycle-level counts as follows (18):

Fertilization rate (%) = (number of 2PN embryos/number of MII oocytes) × 100

Cleavage rate (%) = (number of cleaved embryos/number of 2PN embryos) × 100

Cryopreservation rate (%) = (number of cryopreserved embryos/number of cleaved embryos) × 100

### 2.6 Statistical analysis

Statistical analyses were performed using IBM SPSS Statistics version 25.0 (IBM Corp., Armonk, NY, USA). Continuous variables were assessed for distributional characteristics and are presented as median (minimum–maximum) when analyzed using non-parametric methods. Categorical variables are presented as frequencies and percentages. Comparisons of continuous clinical, semen, and embryological variables among the three paternal age groups (<35, 35–39, and ≥40 years) were performed using the Kruskal–Wallis test. When an overall significant difference was identified, pairwise comparisons were performed with adjustment for multiple comparisons. Categorical variables, including β-hCG positivity, were compared using the Pearson chi-square test or Fisher's exact test, as appropriate.

The associations between paternal age as a continuous variable and embryological parameters were evaluated using Spearman's rank correlation analysis. In addition to the three-group analysis, a secondary analysis comparing paternal age <40 versus ≥40 years was performed using the Mann–Whitney U test for continuous outcomes and the chi-square or Fisher's exact test for categorical outcomes, as appropriate. Multivariable binary logistic regression analysis was performed to evaluate whether paternal age group was independently associated with β-hCG positivity. β-hCG status was coded as negative (0) or positive (1), and the <35-year paternal age group was used as the reference category. The model was simultaneously adjusted for female age, male and female body mass index (BMI), male smoking and alcohol use, baseline anti-Müllerian hormone (AMH), pre-oocyte retrieval (pre-OPU) estradiol (E2), sperm concentration, and sperm motility. Pre-OPU E2 was scaled per 1000-unit increase to facilitate interpretation of the odds ratio. Adjusted odds ratios (aORs) with 95% confidence intervals (CIs) were reported. Model calibration was assessed using the Hosmer–Lemeshow goodness-of-fit test. A sensitivity analysis was additionally performed for the secondary <40 versus ≥40-year comparison of β-hCG positivity using G*Power (version 3.1.9.7), with a two-sided α of 0.05% and 80% power. All statistical tests were two-sided, and a *p*-value <0.05 was considered statistically significant.

## 3 Results

### 3.1 Baseline characteristics of the study population

Baseline clinical and lifestyle characteristics according to paternal age are presented in Table 1. Maternal age differed significantly across paternal age groups, with a median age of 27 years (range, 19–35) in the <35-year group and 32 years in both the 35–39-year (range, 21–35) and ≥40-year (range, 22–35) groups (*p* < 0.001). Sperm concentration and motility were comparable among the paternal age groups (*p* = 0.619 and *p* = 0.845, respectively). No significant between-group differences were observed in infertility duration, male or female BMI, serum AMH, pre-OPU E2, male smoking status, or male alcohol consumption (all *p* > 0.05). Except for maternal age, no statistically significant differences were observed in the evaluated baseline characteristics across paternal age groups.

**Table 1:** Baseline clinical and lifestyle characteristics according to paternal Age.

Characteristic	<35 years (*n* = 129)	35–39 years (*n* = 44)	≥40 years (*n* = 19)	*p*-value
Paternal age, years	30 **(**21–34)	37 **(**35–39)	42 **(**40–54)	—
Maternal age, years	27 **(**19–35)	32 **(**21–35)	32 **(**22–35)	<0**.**001
Infertility duration, years	4 **(**1–14)	5 **(**1–17)	5 **(**1–15)	0**.**061
Male BMI, kg/m^2^	23.94 **(**20.76–28.41)	24.19 **(**20.48–27.74)	23.62 **(**20.24–28.41)	0**.**599
Male smoking, n (%)	28 **(**21.7)	11 **(**25.0)	2 **(**10.5)	0**.**431
Male alcohol consumption, n (%)	17 **(**13.2)	6 **(**13.6)	3 **(**15.8)	0**.**953
Sperm concentration, ×10⁶/mL	40 **(**10–192)	52 **(**2–218)	60 **(**10–136)	0**.**619
Sperm motility, %	40 **(**6–78)	41.5 **(**10–60)	40 **(**8–70)	0**.**845
Female BMI, kg/m^2^	22.48 **(**18.62–30.10)	22.63 **(**18.62–25.88)	21.80 **(**18.83–25.53)	0**.**726
AMH, ng/mL	4.50 **(**2.70–9.40)	4.20 **(**3.00–9.40)	3.80 **(**3.00–8.22)	0**.**358
Pre-OPU E2, pg/mL	2,200 **(**200–17,110)	2,068 **(**734–10,000)	2,300 **(**1,105–7,319)	0**.**935

Data are presented as median (minimum-maximum) or n (%). Continuous variables were compared using the Kruskal-Wallis test and categorical variables using Pearson's chi-square test. Paternal age was not statistically compared because it constituted the grouping variable. BMI, body mass index; AMH, anti-Müllerian hormone; E2, estradiol; OPU, oocyte pick-up.

### 3.2 Embryological characteristics according to β-hCG outcome

Embryological characteristics according to β-hCG outcome are presented in Table 2. Cycles resulting in β-hCG positivity had significantly higher numbers of retrieved oocytes, MII oocytes, 2PN embryos, cleavage-stage embryos, and cryopreserved embryos than β-hCG-negative cycles (all *p* < 0.05). The cryopreservation rate was also significantly higher in β-hCG-positive cycles (*p* = 0.035). In contrast, fertilization and cleavage rates did not differ significantly according to β-hCG outcome (*p* = 0.996 and *p* = 0.100, respectively).

**Table 2:** Comparison of clinical and embryological characteristics according to β-hCG outcome.

Parameter	β-hCG negative (*n* = 80)	β-hCG positive (*n* = 112)	*p*-value
Total oocytes retrieved, n	15 **(**12–53)	16 **(**12–62)	0**.**018
MII oocytes, n	10 **(**2–39)	12 **(**4–37)	0**.**001
2PN embryos, n	9 **(**1–26)	10 **(**3–32)	0**.**004
Fertilization rate, %	83.3 **(**33.3–100)	82.4 **(**31.3–100)	0**.**996
Cleavage-stage embryos, n	8 **(**1–26)	10 **(**2–30)	0**.**001
Cleavage rate, %	100 **(**33.3–100)	100 **(**50.0–100)	0**.**100
Cryopreserved embryos, n	4 **(**1–16)	5 **(**2–25)	<0**.**001
Cryopreservation rate, %	50.0 **(**16.7–100)	56.4 **(**21.4–100)	0**.**035

Data are presented as median (minimum–maximum). Comparisons between β-hCG-negative and β-hCG-positive groups were performed using the Mann–Whitney U test. Bold values indicate *p* < 0.05. MII, metaphase II; 2PN, two pronuclei; β-hCG, beta-human chorionic gonadotropin.

### 3.3 Comparison of embryological outcomes according to paternal Age

Embryological outcomes according to paternal age groups are presented in Table 3. The numbers of retrieved oocytes, MII oocytes, 2PN embryos, cleaved embryos, and cryopreserved embryos did not differ significantly among the <35, 35–39, and ≥40-year paternal age groups (all *p* > 0.05). Similarly, cleavage and cryopreservation rates were comparable among the groups (*p* = 0.484 and *p* = 0.382, respectively). In contrast, the fertilization rate differed significantly among the paternal age groups (*p* = 0.044), with the highest median fertilization rate observed in the 35–39-year group [88.2% (63%–100%)], compared with the <35-year [83.3% (31%–100%)] and ≥40-year [77.8% (56%–100%)] groups. Pairwise analyses showed nominal differences between the <35 and 35–39-year groups (*p* = 0.026) and between the 35–39 and ≥40-year groups (*p* = 0.032); however, neither remained statistically significant after Bonferroni correction (adjusted significance threshold, *p* < 0.0167). No difference was observed between the <35 and ≥40-year groups (*p* = 0.533).

**Table 3:** Embryological outcomes according to paternal age groups.

Outcome	<35 years (*n* = 129)	35–39 years (*n* = 44)	≥40 years (*n* = 19)	*p*-value
Total oocytes retrieved, n	16 **(**12–62)	15 **(**12–29)	17 **(**12–29)	0**.**306
MII oocytes, n	12 **(**2–39)	11 **(**4–20)	11 **(**8–20)	0**.**288
2PN embryos, n	10 **(**1–32)	10 **(**3–19)	10 **(**5–15)	0**.**812
Fertilization rate, %	83.3 **(**31–100)	88.2 **(**63–100)	77.8 **(**56–100)	0**.**044
Cleavage-stage embryos, n	9 **(**1–30)	9 **(**3–19)	10 **(**5–15)	0**.**760
Cleavage rate, %	100 **(**33–100)	100 **(**64–100)	100 **(**73–100)	0**.**484
Cryopreserved embryos, n	5 **(**1–25)	5 **(**1–12)	6 **(**2–10)	0**.**110
Cryopreservation rate, %	50.0 **(**20–100)	55.6 **(**17–100)	63.6 **(**36–89)	0**.**382

Data are presented as median (minimum–maximum). Comparisons among paternal age groups were performed using the Kruskal–Wallis test. When an overall significant difference was identified, pairwise comparisons were performed using the Mann–Whitney U test with Bonferroni correction (adjusted significance threshold, *p* < 0.0167). Rates are expressed as percentages. Bold values indicate *p* < 0.05 in the overall Kruskal–Wallis test. MII, metaphase II; 2PN, two pronuclei.

### 3.4 Pregnancy outcome according to paternal age

β-hCG positivity was observed in 82 of 129 cycles (63.6%) in the <35-year paternal age group, 20 of 44 cycles (45.5%) in the 35–39-year group, and 10 of 19 cycles (52.6%) in the ≥40-year group (Table 4). Although the proportion of β-hCG-positive cycles was highest in the <35-year group, the difference among the three paternal age groups was not statistically significant (*χ*^2^ = 4.710, df = 2, *p* = 0.095).

**Table 4:** β-hCG positivity according to paternal age groups.

Outcome	<35 years (*n* = 129)	35–39 years (*n* = 44)	≥40 years (*n* = 19)	*p*-value
β-hCG negative, n (%)	47 (36.4)	24 (54.5)	9 (47.4)	0.095
β-hCG positive, n (%)	82 (63.6)	20 (45.5)	10 (52.6)	

Data are presented as n (%). Comparisons among paternal age groups were performed using the Pearson chi-square test. β-hCG, beta-human chorionic gonadotropin.

### 3.5 Multivariable analysis of β-hCG positivity

In multivariable logistic regression analysis adjusted for female age, male and female BMI, male smoking and alcohol use, baseline AMH, pre-OPU E2, sperm concentration, and sperm motility, paternal age group was not independently associated with β-hCG positivity (overall *p* = 0.358) (Table 5). Compared with men aged <35 years, the adjusted odds of β-hCG positivity were not significantly different in the 35–39-year group (aOR=0.574, 95% CI: 0.268–1.227; *p* = 0.152) or the ≥40-year group (aOR=0.813, 95% CI: 0.288–2.298; *p* = 0.697). None of the additional covariates, including sperm concentration and motility, were independently associated with β-hCG positivity (all *p* > 0.05). The overall model was not statistically significant (*χ*^2^ = 7.532, df = 11, *p* = 0.755), and the Hosmer–Lemeshow test indicated no evidence of poor model fit (*p* = 0.985).

**Table 5:** Multivariable logistic regression analysis for β-hCG positivity.

Variable	aOR	%95 CI	*p*-value
Paternal age group, overall	—	—	0**.**358
35–39 vs. <35	0**.**574	0.268**–**1.227	0**.**152
≥40 vs. <35	0**.**813	0.288**–**2.298	0**.**697
Female age, per year	0**.**956	0.885**–**1.032	0**.**250
Male BMI, kg/m^2^	0**.**956	0.778**–**1.174	0**.**667
Female BMI, kg/m^2^	1**.**041	0.908**–**1.192	0**.**564
Male smoking,	1**.**288	0.609**–**2.726	0**.**508
Male alcohol	0**.**941	0.395**–**2.242	0**.**890
AMH	1**.**019	0.854**–**1.215	0**.**838
Pre-OPU E2, per 1,000	1**.**034	0.897**–**1.191	0**.**643
Sperm concentration, per 10⁶/mL	1**.**000	0.992**–**1.007	0**.**915
Motility, per 1% increase	0**.**994	0.974**–**1.015	0**.**578

aOR, adjusted odds ratio; CI, confidence interval; BMI, body mass index; AMH, anti-Müllerian hormone; E2, estradiol. The dependent variable was β-hCG positivity (negative=0, positive=1). Paternal age <35 years was used as the reference category. Pre-OPU E2 was scaled per 1,000-unit increase. The model was simultaneously adjusted for female age, male and female BMI, male smoking and alcohol use, baseline AMH, pre-OPU E2, sperm concentration, and sperm motility. Model statistics: *χ*^2^ = 7.532, df = 11, *p* = 0.755; Nagelkerke R^2^ = 0.052; Hosmer–Lemeshow *χ*^2^ = 1.849, df = 8, *p* = 0.985.

## 4 Discussion

The present study evaluated the association between paternal age and embryological and early pregnancy outcomes in women with PCOS undergoing ICSI. The main finding was that paternal age was not consistently associated with impaired embryological development or β-hCG positivity. Although the overall fertilization rate differed among paternal age groups, this finding was not supported by the *post hoc* pairwise comparisons after correction for multiple testing. Furthermore, the numbers of retrieved oocytes, MII oocytes, 2PN embryos, cleavage-stage embryos, and cryopreserved embryos, as well as cleavage and cryopreservation rates, were comparable among paternal age groups. s-hCG positivity also did not differ significantly according to paternal age, and paternal age remained unrelated to this outcome after adjustment for maternal and paternal characteristics, ovarian reserve and stimulation-related variables, and conventional semen parameters.

The effect of paternal age on ART outcomes remains controversial. A systematic review and meta-analysis by Murugesu et al. (19). suggested that younger paternal age may be associated with more favorable blastocyst and miscarriage outcomes; however, substantial heterogeneity existed among studies with respect to paternal age thresholds, treatment characteristics, and control of maternal factors. Similarly, Gourinat et al. (8) concluded that although advancing paternal age is associated with biological changes in male reproductive function, evidence regarding its independent effect on ART outcomes remains inconsistent. Large contemporary cohort studies further indicate that maternal age and ART modality may modify the apparent effect of paternal age. Datta et al. (20), in an analysis of 59,951 fresh IVF/ICSI cycles, found that the association between paternal age and live birth was particularly evident in IVF cycles involving women aged 35–39 years, whereas a similar detrimental association was not demonstrated among women younger than 35 years or in ICSI cycles. Marsidi et al. (21), analyzing more than 77,000 fresh IVF cycles, likewise demonstrated that paternal-age associations varied according to maternal age. These observations are particularly relevant to our cohort, in which all women were ≤35 years and treatment was performed using ICSI.

Our fertilization findings deserve specific consideration. The overall fertilization rate differed among paternal age groups, with the highest median value in the 35–39-year group and the lowest in the ≥40-year group. However, the absence of significant pairwise differences after Bonferroni correction and the lack of a monotonic age-related decline argue against interpreting this finding as evidence of a clear paternal-age effect. Previous findings regarding fertilization are similarly inconsistent. Elbardisi et al. (22) reported poorer normal fertilization and cleavage outcomes with increasing paternal age in an ICSI population, despite finding no corresponding differences in clinical pregnancy, miscarriage, or live birth. Collectively, these findings suggest that early embryological endpoints may be sensitive to paternal characteristics in some populations (23, 24), but the magnitude and clinical relevance of these associations remain uncertain. In our cohort, the isolated overall fertilization difference should therefore be interpreted cautiously rather than as evidence of progressive age-related impairment.

Importantly, conventional semen parameters were comparable among the paternal age groups in the present study. Neither sperm concentration nor motility differed significantly with paternal age, and neither parameter independently predicted β-hCG positivity in the adjusted model. Nevertheless, the absence of differences in conventional semen parameters does not exclude age-related changes in sperm functional or genomic integrity. Aging has been associated with increased oxidative stress, sperm DNA fragmentation, impaired chromatin integrity, accumulation of *de novo* mutations, and epigenetic alterations (25, 26). A systematic review by Gonzalez et al. (12) found that most included studies demonstrated an increase in sperm DNA fragmentation with advancing paternal age. Current evidence therefore suggests that chronological aging may affect sperm characteristics that are not captured by routine measurements of concentration and motility. Because sperm DNA fragmentation, oxidative stress, chromatin integrity, and genetic or epigenetic alterations were not assessed in our cohort, subtle age-related sperm dysfunction cannot be excluded.

The restriction of the study population to women with PCOS should also be considered when interpreting these findings. PCOS represents a heterogeneous reproductive and metabolic disorder in which endocrine abnormalities, ovarian response, oocyte competence, and the metabolic environment may influence ART outcomes (27, 28). Contemporary reviews emphasize that ART outcomes in PCOS are shaped by multiple interacting ovarian and metabolic factors (29). Thus, although focusing on a PCOS population improved clinical homogeneity, it may also limit the generalizability of our findings to women undergoing ICSI for other infertility indications (29, 30). Moreover, maternal age differed significantly among paternal age groups in our cohort, reflecting the expected correlation between partners' ages. This potential confounding was addressed by including female age in the multivariable model, in which paternal age remained unrelated to β-hCG positivity (31, 32).

Several limitations should be acknowledged. First, the retrospective and single-center design limits causal inference and external generalizability. Second, the ≥40-year paternal age group included only 19 cycles, resulting in relatively wide confidence intervals and limited statistical power to detect modest associations. A sensitivity analysis for the <40 versus ≥40-year comparison indicated that, given the available group sizes (*n* = 173 vs. *n* = 19) and a β-hCG positivity rate of 59.0% in the <40-year group, the study had 80% power at a two-sided *α* of 0.05 to detect only a relatively large difference, corresponding to a β-hCG positivity rate of approximately 25.6% or lower in the ≥40-year group. Therefore, smaller or moderate paternal-age effects may have remained undetected, and the absence of statistical significance should not be interpreted as evidence of equivalence. Third, β-hCG positivity was the available pregnancy endpoint. International ART terminology distinguishes biochemical pregnancy from clinical pregnancy, ongoing pregnancy, and live birth; therefore, β-hCG positivity cannot establish whether paternal age influences later reproductive outcomes. Importantly, the absence of an association with β-hCG positivity does not exclude a potential paternal-age effect at subsequent stages of pregnancy, including early pregnancy loss, fetal viability, or live birth. Paternal age-related alterations in sperm genomic or epigenetic integrity may have consequences that are not fully captured by fertilization, early embryo development, or initial β-hCG detection. Therefore, the present findings should be interpreted as being limited to early reproductive outcomes and should not be extrapolated to overall reproductive success. Future prospective studies with systematic follow-up through ultrasound-confirmed clinical pregnancy, miscarriage, ongoing pregnancy, and live birth are required to determine the clinical significance of paternal age in this population. Fourth, detailed sperm functional assessments, including DNA fragmentation, oxidative stress, chromatin integrity, and genetic or epigenetic analyses, were unavailable. Finally, although the multivariable model accounted for female age, male and female BMI, smoking, alcohol consumption, AMH, pre-OPU E2, sperm concentration, and sperm motility, residual confounding inherent to retrospective studies cannot be excluded.

Despite these limitations, the present study provides data on paternal age within a clinically defined PCOS population undergoing ICSI. The inclusion of conventional semen parameters and several maternal and paternal covariates in the adjusted analysis allowed the association between paternal age and early pregnancy outcome to be evaluated beyond chronological age alone. Overall, our findings do not demonstrate a consistent detrimental association between increasing paternal age and the evaluated embryological outcomes or β-hCG positivity. Rather, they support the view that the reproductive implications of paternal aging are likely to be context-dependent and may not be adequately characterized by chronological age or conventional semen parameters alone. Larger studies incorporating sperm functional and molecular markers and clinically meaningful endpoints such as clinical pregnancy, miscarriage, ongoing pregnancy, and live birth are needed to clarify the independent contribution of paternal age to ICSI outcomes, particularly in men aged ≥40 years.

## 5 Conclusion

In this cohort of women with PCOS undergoing ICSI, paternal age was not independently associated with β-hCG positivity or with a consistent deterioration in embryological outcomes. Although fertilization rate differed among paternal age groups in the overall analysis, this difference was not supported by significant pairwise comparisons after correction for multiple testing. Other embryological outcomes, including oocyte yield, MII oocyte number, 2PN embryos, cleavage-stage embryos, cryopreserved embryos, cleavage rate, and cryopreservation rate, were comparable across paternal age groups. These findings suggest that chronological paternal age alone may have limited predictive value for early ICSI outcomes in this population. However, the small number of men aged ≥40 years and the absence of sperm functional and molecular assessments and later pregnancy outcomes warrant cautious interpretation. Larger prospective studies incorporating sperm DNA integrity and clinically relevant endpoints, particularly live birth, are needed to better define the reproductive impact of advanced paternal age.

## Institutional review board statement

The study was conducted in accordance with the Declaration of Helsinki and approved by the Çukurova University Faculty of Medicine Research Ethics Committee (Meeting No: 161, Decision No: 60, Date: December 5, 2025).

## Data Availability

The original contributions presented in the study are included in the article/Supplementary Material, further inquiries can be directed to the corresponding author.
